# Patiromer Acetate Induced Hypercalcemia: An Unreported Adverse Effect

**DOI:** 10.1155/2019/3507407

**Published:** 2019-02-04

**Authors:** Shreeyukta Bhattarai, Stephen Pupillo, Gulshan Man Singh Dangol, Erdal Sarac

**Affiliations:** ^1^Northeast Ohio Medical University, Department of Medicine, Rootstown, Ohio, USA; ^2^St. Elizabeth Youngstown Hospital, Department of Internal Medicine, Youngstown, Ohio, USA; ^3^Lake Erie College of Osteopathic Medicine, Erie, Pennsylvania, USA; ^4^Ohio University Heritage College of Osteopathic Medicine, Athens, Ohio, USA

## Abstract

Hyperkalemia, a potential life threating condition, is a commonly encountered problem in chronic kidney disease (CKD) patients. Patiromer acetate, a nonabsorbable cation exchange polymer, is a gastrointestinal agent for chronic therapy in patients with persistent hyperkalemia. Patiromer is generally well tolerated in patients; common side effects are gastrointestinal, such as diarrhea, constipation, flatulence, and vomiting. Hypercalcemia, although a theoretical possibility, has not been reported in any major clinical trials. We present a case of hypercalcemia associated with patiromer acetate used for treatment of hyperkalemia in a stage IV CKD patient. Clinicians should be aware of the possibility of hypercalcemia while taking patiromer.

## 1. Introduction

Patiromer acetate, a nonabsorbable cation exchange polymer, was recently approved for chronic management of hyperkalemia. Patiromer acetate decreases serum potassium by exchanging calcium for potassium in the intestine, especially the colon, resulting in gastrointestinal loss of potassium. It is considered safe and well tolerated; gastrointestinal side effects include diarrhea, constipation, flatulence, and vomiting. Other potential side effects include hypomagnesemia and hypokalemia. We present a case of a seventy-year-old man with diabetes, CKD stage IV, and hypertension with hypercalcemia on patiromer acetate for consistently elevated potassium.

## 2. Case

During routine follow-up, a 70-year-old Caucasian male with past medical history of type 2 diabetes mellitus, gout, chronic kidney disease (CKD) stage IV, anemia of chronic disease, vitamin D deficiency, and hypertension, managed with patiromer acetate for persistent hyperkalemia secondary to CKD, presented with hypercalcemia. Home medications included metformin, allopurinol, weekly erythropoietin, and vitamin D supplementation. Serum potassium was persistently above 5.5 mmol/L prior to treatment initiation. Estimated glomerular filtration rate (eGFR) was 24 ml/min/1.73 m^2^, blood urea nitrogen (BUN) was 86 mg/dl, and creatinine was 2.6 mg/dl. Other labs included calcium (Ca), 9.2 mg/dl; potassium (K), 5.7 mmol/L; and parathyroid hormone (PTH), 86 pg/ml. BUN and creatinine were similar over the last year. Initial patiromer acetate dosing was 8.5 mg nightly. Symptomatically, the patient tolerated the medication very well. However, calcium at 30-day follow-up increased to 10.2 mg/dl, and potassium level decreased to 5.1 mmol/L. Since the patient was asymptomatic, he was advised to continue patiromer acetate and discontinue vitamin D supplementation. Repeat lab values after two months demonstrated higher calcium, 10.7 mg/dl, and unchanged potassium, 5.1 mmol/L.

At this point, secondary causes of hypercalcemia were investigated. See Table 1. Mild hyperparathyroidism of 86pg/ml before the initiation of therapy (normal 15-65pg/ml) was considered secondary to vitamin D deficiency. 25-hydroxy (OH) Vitamin D was 31 ng/ml (normal: 30-100ng/ml), and 1, 25-OH Vitamin D was 10.2 pg/ml (normal: 19.9-79.3pg/ml), suggesting insufficient 1-alpha hydroxylase enzyme secondary to CKD. Parathyroid hormone related peptide (PTHrP) was within the normal limit, 2.1 pmol/L (normal: 0.0-2.3pmol/L). Normal bone density was observed on dual energy X-ray absorptiometry (DEXA) scan; the lowest T score (– 1.2), was femoral. Urinalysis was negative for proteinuria; urine immunofixation demonstrated no light chains. Thyroid stimulating hormone (TSH) level was 0.874 uIU/mL (normal 0.27-4.2uIU/mL). Chest computed tomography (CT) scan showed multiple bilateral 2-3 mm calcified and noncalcified pulmonary nodules. Nodules were stable in size, compared to scan seven years before, and considered noncontributory to hypercalcemia. Angiotensin converting enzyme (ACE) level was 53 U/L (normal 9-67 U/L).

**Figure 1:**
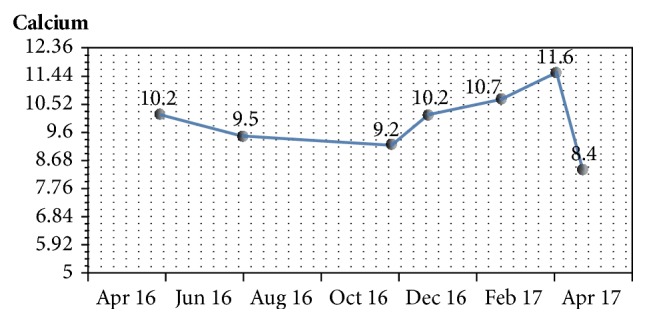
Trend in calcium level during the course of treatment with patiromer acetate.

**Figure 2:**
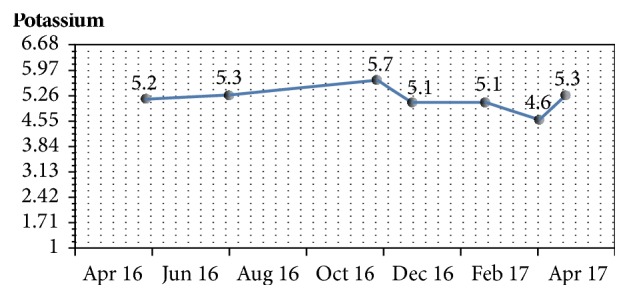
Trend in potassium level during the course of treatment with patiromer acetate.

**Figure 3:**
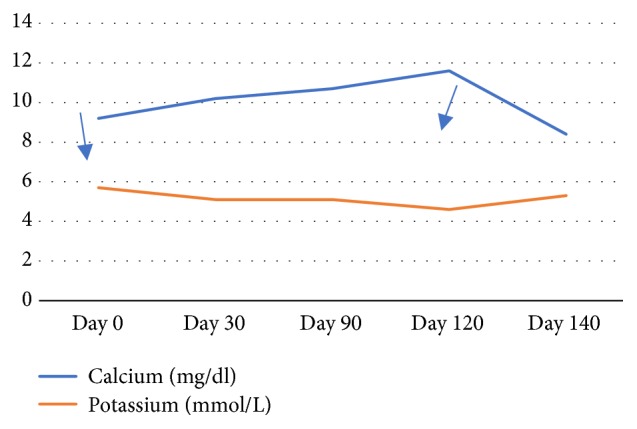
Trend in calcium and potassium level during the course of treatment with patiromer acetate. Arrows indicate start and stop date of patiromer acetate.

With no obvious secondary causes of hypercalcemia on laboratory assessment and imaging, patiromer was discontinued. Despite discontinuation, he continued the medication because of misunderstanding. On follow-up after an additional 30 days, calcium returned even higher, 11.6 mg/dL (Figure 1), and potassium even lower, 4.6 mmol/L (Figures 2 and 3). After additional patient education, he was advised again to stop taking patiromer acetate. One month after stopping medication, calcium normalized to 8.4 mg/dL. PTH level, suppressed at 10 pg/ml during the hypercalcemic state, returned to 66 pg/ml after calcium normalization and patiromer acetate cessation. BUN, 80 mg/dl; creatinine, 2.8 mg/dl; and GFR, 22 mL/min/1.73 m^2^ remained stable. Potassium trended upward, 5.3 mmol/L.

## 3. Discussion

Prevalence of hyperkalemia in CKD patients is directly proportional to the residual renal function and increases from 13% in CKD stage II to 34% in CKD stage IV.[1] Gastrointestinal tract and renal systems are important regulators of potassium homeostasis. In patients with intact renal function, approximately 90 percent of potassium is excreted through the kidneys, the remaining 10 percent excreted through the gastrointestinal tract, especially the colon.[2] Renal potassium excretion is controlled by different physiologic signals, such as aldosterone, sodium delivery in the distal tubule, urine output, dietary potassium, and acid base status. Additional potassium is processed by the colon through passive and active routes. Excretion occurs paracellularly through the passive route, whereas active secretion occurs through BK channels, also known as Big Potassium channels, present on the apical surface of colonic epithelial cells. When urinary potassium excretion is decreased in CKD patients, both active and passive mechanisms for colonic potassium excretion are upregulated, resulting in increased potassium excretion through the gastrointestinal tract.[3] However, adaptive responses may be insufficient to prevent hyperkalemia. Other contributing causes of hyperkalemia in CKD patients include use of angiotensin converting enzyme (ACE) inhibitors, angiotensin receptor blockers (ARBs), aldosterone antagonist, and renin inhibitors.

Patients with chronic mild to moderate hyperkalemia not requiring urgent lowering of serum potassium can be treated with dietary modification, diuretics, and discontinuation of offending medications. Cation exchange resins, such as sodium polystyrene sulfonate (SPS), approved for hyperkalemia more than 50 years ago, did not gain popularity because of side effects, such as fluid retention and bowel necrosis. [4] Patiromer acetate, a nonabsorbable cation exchange polymer, was recently approved as a gastrointestinal agent for chronic therapy in patients with persistent hyperkalemia. [5]

Patiromer acetate binds and exchanges potassium for calcium in the gastrointestinal tract, especially the colon. Potassium is the most abundant cation in the colon, and patiromer remains for the longest period in this segment of the gastrointestinal tract. Although potassium exchange can occur in the proximal gastrointestinal tract, colonic pH causes ionization of the patiromer molecule resulting in maximum ion exchange and increased potassium binding. Moreover, abundant colonic potassium, especially in CKD patients where BK channels are upregulated, makes patiromer more effective. Additionally, by binding potassium in the colonic lumen, patiromer creates a concentration gradient, increasing potassium secretion via BK channels.[3] Patiromer acetate demonstrates significant potassium lowering effect in patients with heart failure with concomitant renin-angiotensin-aldosterone inhibitor therapy, in diabetic nephropathy and in CKD patients. [6–8]

Bushinsky et al. studied the relationship between urinary excretion of ions and patiromer at various doses and suggested that any change in urinary ion excretion would reflect corresponding change in GI absorption of that ion. He noted a small, 73mg/day, increase in urinary calcium excretion, when healthy volunteers ingesting a normal 1000mg/day calcium containing diet used the maximum FDA approved dose of patiromer, 8.4 gm, three times daily. This suggested that only a small fraction of the calcium released from patiromer was absorbed.[9] Calcium released from patiromer in exchange for potassium can have multiple fates: it can bind to phosphorous and/or oxalate forming insoluble calcium phosphate and/or calcium oxalate salts and be excreted in the stool; it can rebind with patiromer; or it can be systematically absorbed. [9, 10] In a yearlong study of diabetic patients with GFR of 15- 59 ml/min/m2 treated with variable doses of patiromer, no clinically significant changes in serum calcium were noted.[7] At baseline calcium was 9.4-9.6 mg/dl across the patiromer dosing groups, and at weeks 4, 28, and 52, mean change in calcium from baseline was not statistically significant. The current patient, CKD stage IV and diabetic, was similar to patients studied by Bakris et al. but exhibited hypercalcemia with patiromer treatment and resolution on discontinuation. We suspect that patiromer use in this patient may have been associated with increased systematic calcium absorption, reduced renal calcium excretion, and/or a problem with systematic buffering of an exogenous calcium load in our patient. While there is no literature on drug-drug interaction, vitamin D supplementation with patiromer may play a role in hypercalcemia development. However, of note, serum calcium in the current patient continued to rise after vitamin D cessation. Although the mechanism remains unclear, some patients may be more prone to adverse effects. Discovery of these factors will require further investigation.

## 4. Conclusion

Although colonic excretion of potassium is increased in CKD patients, it may be inadequate to maintain normal levels. Patiromer is relatively safe and effective for hyperkalemia in CKD patients. Hypercalcemia has not been reported in any major clinical trials. Our case report of patiromer-induced hypercalcemia reminds clinicians of the importance of monitoring patient response in new treatment regimens, as well as the need for diligent investigations into urinary and serum calcium.

## Figures and Tables

**Table 1:** 

	Day 1	Day 30	Day 90	Day 120	Day 150
Potassium, mmol/L	5.7	5.1	5.1	4.6	5.3
eGFR, ml/min/m2	24	23	31	25	22
BUN, mg/dL	86	92	65	87	80
Creatinine, mg/dL	2.6	2.7	2.1	2.5	2.8
Calcium, mg/dL	9.2	10.2	10.7	11.6	8.4
PTH, pg/ml	86			10	204
25 OH vitamin D, ng/mL	31				34
